# Zootechnical Additives Associated with Antimicrobials: Effects on Immune Response and Intestinal Histomorphometry in Broiler Chickens

**DOI:** 10.3390/vetsci12060581

**Published:** 2025-06-13

**Authors:** Kenes Leonel de Morais Castro, Nilton Rohloff Júnior, Elaine Talita Santos, Jean Kaique Valentim, Rodrigo Garófallo Garcia, Giancarlo Rieger, Sarah Sgavioli

**Affiliations:** 1 Postgraduate Program in Animal Production (PMPPA), Brazil University, Av. Hilário de Silva Passos, 950, Parque Universitário, Descalvado, São Paulo 13690-000, Brazil; kenesvet22@gmail.com (K.L.d.M.C.); gian_rieger@hotmail.com (G.R.); 2 Postgraduate Program in Animal Science (PPGCA), Western Paraná State University (UNIOESTE), Rua Pernambuco, 1777, Centro, Marechal Cândido Rondon 85960-000, Brazil; nilton.junior2@unioeste.br; 3 Postgraduate Program in Animal Science (PPGCA), São Paulo State University (UNESP), Access Route Prof. Paulo Donato Castellane, s/n, Jaboticabal, São Paulo 14884-900, Brazil; elainesantost20@gmail.com; 4 Department of Animal Production (DPA), Institute of Animal Science, Federal Rural University of Rio de Janeiro (UFRRJ), Zona Rural, BR-465, Km 7, Seropédica, Rio de Janeiro 23890-000, Brazil; jean.valentim@ufrrj.br; 5 Faculty of Agricultural Sciences (FCA), Federal University of Grande Dourados (UFGD), Rodovia Dourados-Itahum, Km 12, Cidade Universitária, Dourados 79804-970, Brazil; rodrigogarcia@ufgd.edu.br; 6 Faculdade Marechal Rondon (FARON), Avenida Marechal Rondon, 100058, Setor Industrial, Vilhena 76980-000, Brazil; 7 Graduate Program in Animal Science (PPGCA), Pontifícia Universidade Católica do Paraná (PUCPR), Rua Imaculada Conceição, 1155, Prado Velho, Curitiba 80215-901, Brazil

**Keywords:** organic acid, essential oil, prebiotic, probiotic, intestinal health

## Abstract

This study evaluated the effects of zootechnical additives in combination with antibiotic growth promoters (AGPs) on broiler performance, leukocyte differential activity, phagocytic activity, intestinal histomorphometry, and the inside index. A total of 1400 one-day-old Cobb 500 male chicks were assigned to a completely randomized design with seven treatments and eight replicates of 25 birds per unit. The treatments included a basal diet without AGPs, a basal diet with AGPs, and AGPs combined with different zootechnical additives: prebiotics (fructooligosaccharides, galactooligosaccharides, beta-glucans, and mannan-oligosaccharides), probiotics (*Bacillus subtilis* LFU160), essential oils (cashew nut shell liquid), and organic acids (butyric acid glycerides). The results showed the treatments combining AGPs and zootechnical additives had better effects (*p* < 0.05) on weight gain, feed intake, the feed conversion ratio, and the productive efficiency index. The phagocytic activity was worse in birds without AGPs and with AGPs but without additives. However, birds that received AGPs in combination with zootechnical additives exhibited a poorer intestinal histomorphometry and lower inside index compared to those fed only AGPs or diets without AGPs. In conclusion, zootechnical additives can be used alongside AGPs to enhance broiler performance and immune function, particularly during early growth stages, with essential oils showing the most promising results.

## Simple Summary

Antibiotic growth promoters (AGPs) are commonly used in poultry farming to enhance growth and health, but concerns about antibiotic resistance have driven the search for alternatives. This study investigated the effects of combining AGPs with different zootechnical additives, including prebiotics, probiotics, essential oils, and organic acids, on broiler chicken performance, immune response, and gut health. A total of 1400 chicks were divided into seven feeding groups, and their growth, feed efficiency, immune activity, and intestinal structure were evaluated. The results showed that zootechnical additives combined with AGPs improved the broiler chickens’ growth performance compared to AGPs alone, but some combinations negatively affected their gut structure. Essential oils were particularly effective, supporting both their growth and immune function. These findings provide valuable insights for poultry producers seeking to optimize feed strategies while reducing the reliance on antibiotics.

## 1. Introduction

The incorporation of zootechnical additives in broiler diets is a strategy that not only reduces costs but also enhances poultry productivity [1]. These additives, which can either replace or be used in conjunction with antibiotic growth promoters (AGPs), contribute to weight gain and improved feed efficiency [2].

Among these additives, organic acids have gained prominence as a viable alternative for balancing the intestinal microbiota and promoting animal welfare [3]. Recognized as safe for use in animal nutrition, organic acids can be administered individually or in combination, showing beneficial effects in reducing pathogenic microorganisms in animals’ intestines [4].

Another important group of additives is essential oils, which are aromatic compounds extracted from various plant parts. These bioactive substances exhibit a range of biological effects. Several plants, including cinnamon (*Cinnamomum* spp.), oregano (*Origanum* spp.), orange (*Citrus sinensis* L.), sage (*Salvia officinalis* L.), rosemary (*Rosmarinus officinalis* L.), and cashew trees (*Anacardium occidentale* L.), have been identified as potential supplements for broiler chickens [5].

In addition, probiotics and prebiotics play crucial roles in regulating intestinal immunity and modulating the mRNA expression of junction proteins in broiler chickens [6]. Studies suggest that dietary supplementation with probiotic blends can significantly enhance intestinal barrier function. Probiotics have demonstrated their ability to adhere to the intestinal epithelium, resist acidic conditions, and competitively antagonize and eliminate certain pathogens in vivo [7].

Nutrition plays a vital role in regulating the immune system of poultry, as its proper function depends on the availability of energy and essential nutrients required for the formation of immune cells and other defense-related substances [8]. Consequently, immunonutrition has gained increasing importance and is defined as the ability to increase the body’s resistance to diseases through the use of immunomodulatory nutrients [4]. Zootechnical additives such as probiotics and acidifiers often lead to beneficial changes in intestinal morphology and overall poultry performance [9].

However, some conflicting and inconsistent findings have raised concerns regarding the precise mechanisms or modes of action of probiotics and acidifiers, highlighting a gap in scientific knowledge [10]. It is believed that probiotics influence not only local mucosal immunity but also systemic immune responses. Phagocytosis, for example, triggers the release of a wide array of chemical mediators, leading to the recruitment of immunocompetent cells and the development of a systemic immune response [11]. During the first weeks of life, the activation of lymphoid tissues enhances nonspecific immune responses, which contribute to overall immunity and even improve vaccine efficacy [12].

Considering the importance of zootechnical additives, their inclusion in poultry diets can support both the integrity and development of the intestinal mucosa [13]. This results in improved digestibility, enhanced nutrient absorption, and, consequently, better overall performance and product quality [14].

Given the extensive evidence supporting the benefits of zootechnical additives, further research is essential to expand our knowledge of their positive effects on intestinal health and immune modulation [3]. This includes evaluating the inside index, intestinal histomorphometry in the small intestine, phagocytic activity, and leukocyte differential counts in broiler chickens.

Hematological parameters serve as key indicators of an animal’s health status and are critical for diagnosing pathologies. Variations in these parameters may signal adverse reactions to specific dietary additives, inadequate management, or disease outbreaks within poultry flocks. For example, an increased leukocyte count is often associated with the body’s immune response to infection [11].

Thus, the objective of this study was to evaluate the effects of zootechnical additives, prebiotics, probiotics, essential oils, and organic acids when combined with AGPs on the performance, leukocyte differential, phagocytic activity, inside index, and intestinal histomorphometry of broiler chickens.

## 2. Materials and Methods

### 2.1. Facility, Birds, and Management

The experiment was conducted in a commercial poultry house located in Guapiaçu, São Paulo, Brazil. The study was approved by the Animal Ethics Committee of Universidade Brazil under protocol number 21000004.

A total of 1400 male Cobb 500 broiler chicks, with an initial average weight of 43.25 ± 0.2 g, were obtained from the Avozeiro Industrial Hatchery [15] and reared from 1 to 44 days of age. The birds were housed in 56 wire-meshed pens made of PVC pipes, each measuring 1.20 m × 2.00 m (2.40 m^2^), arranged along the inner side of a conventional poultry house (12 m × 72 m; 864 m^2^) equipped with a positive pressure ventilation system, including fans and misting sprinklers. The stocking density was 10.41 birds/m^2^ (25 birds per pen), and the litter consisted of fresh pine shavings at a depth of 10 cm.

Throughout the trial, the birds had *ad libitum* access to feed and water and were managed according to the lighting, temperature, humidity, and husbandry recommendations outlined in the Cobb Broiler Management Guide [15]. Air temperature and relative humidity were monitored via three digital thermohygrometers distributed evenly within the facility. The average weekly temperatures recorded from the first to the sixth week were 27.1 °C, 28.3 °C, 25.8 °C, 24.2 °C, 23.8 °C, and 23.2 °C, respectively, while the corresponding relative humidity values were 37.4%, 67.8%, 67.8%, 67.8%, 60.8%, and 58.5%, respectively.

The lighting program followed the guidelines for the Cobb strain [15] and was adjusted as follows: 24 h of light on the first day, 23 h from days 2 to 7, 20 h from days 8 to 14, 18 h from days 15 to 21, and 14 h from days 22 to 44. Chicks were vaccinated against Marek’s disease at the hatchery. Upon arrival, they received an infectious bronchitis vaccine via spray application, followed by vaccination against infectious bursal disease (Gumboro) at seven days of age. Booster doses for infectious bronchitis and Gumboro were administered by drinking water at 14 days of age.

The experimental period was divided into four feeding phases: pre-starter (1 to 8 days), starter (9 to 21 days), grower (22 to 38 days), and finisher (39 to 44 days). The performance and physiological parameters were evaluated throughout the entire rearing period from 1 to 44 days of age.

### 2.2. Experimental Design

The birds were distributed in a completely randomized design consisting of seven treatments (Table 1), with eight replicates per treatment and 25 birds per experimental unit.

### 2.3. Experimental Diets

The diets, in mash form, were based on corn and soybean meal and were formulated according to the nutritional recommendations of Rostagno et al. [16] for each production phase. Zootechnical additives were included “on top” of the diets according to the respective treatments. The proximate composition and calculated nutritional values of the experimental diets are presented in Table 2.

### 2.4. Evaluated Parameters

#### 2.4.1. Performance

Body weight gain (BWG), feed intake (FI), and the feed conversion ratio (FCR) were evaluated in the following phases: pre-starter (1 to 8 days), starter (9 to 21 days), grower (22 to 39 days), finisher (40 to 44 days), and overall (1 to 44 days). Livability (LIV) and the productive efficiency index (PEI) were assessed only for the overall rearing period.

Body weight gain was calculated as the difference between the final weight and the initial weight at the end of each phase. Feed intake was determined by the difference between the total feed provided and the residual feed at the end of each phase. The feed conversion ratio was calculated as the total feed intake divided by weight gain during the period. Both FI and FCR were adjusted for mortality [17].

Livability (LIV) was determined on the basis of the cumulative mortality from 1 to 44 days of age. The productive efficiency index (PEI) was calculated via the following formula:

PEI = [BWG (g) × LIV (%]/(FCR × 10).

#### 2.4.2. Leukocyte Differential

Leukocyte differential counts were performed for lymphocytes, heterophils, monocytes, eosinophils, and basophils at 6 and 21 days of age in one bird per experimental unit, totaling eight birds per treatment. Blood samples were collected from the brachial vein and stored in plastic tubes containing 15 µL of heparin per 1 mL of blood (Glistab, cat. 29, Labtest Diagnóstica) on ice. Blood smears were prepared immediately after collection. After air drying, the smears were stained via a Panoptic staining kit, rapidly dehydrated in an increasing ethanol concentration series, cleared with xylene, and mounted with Entellan [18].

The percentages of different leukocyte types (lymphocytes, heterophils, monocytes, eosinophils, and basophils) were determined by counting 100 leukocytes per bird. Differential leukocyte counts are expressed as cells/mm^3^ [19]. The heterophil-to-lymphocyte (H/L) ratio was calculated for each bird. Total leukocyte counts (leukocytes/mm^3^) were determined using a Neubauer chamber immediately after blood collection, with samples diluted (1:300) in 0.01% toluidine blue solution.

#### 2.4.3. Phagocytic Activity, Intestinal Histomorphometry, and the Inside Index

Blood samples were collected at 6 and 21 days of age, with one bird per pen, for a total of eight birds per treatment. Blood was drawn via cardiac puncture via sterile syringes and needles following standard animal restraint and aseptic protocols [20]. After collection, 3 mL aliquots of blood were placed in 10 cm nonvacuum test tubes containing anticoagulant (EDTA) and analyzed via flow cytometry, for which mononuclear cells were separated from other blood components and subsequently labeled with specific antibodies.

Mononuclear cells were isolated via density gradient centrifugation with Ficoll (Histopaque-1077^®^, (Sigma-Aldrich, St. Louis, MO, USA)) following the protocol described by Fair et al. [21]. Briefly, blood was diluted 1:1 in phosphate-buffered saline (PBS, pH 7.4) to a final volume of 2 mL, carefully layered over 1 mL of Ficoll in a sterile 15 mL conical tube, and centrifuged (Eppendorf 5804 R) at 400× *g* for 30 min at room temperature. The resulting buffy coat was transferred to a new tube, washed twice with 4 mL of PBS, and centrifuged at 400× *g* for 7 min. The final pellet was resuspended in 1 mL of 1% paraformaldehyde solution in PBS for cell fixation. Thirty minutes after resuspension, the cells were washed, centrifuged, and resuspended in PBS with 1% bovine serum albumin (BSA; Sigma-Aldrich). Cell counts were performed using a Neubauer chamber (NewOptics).

Fluorescence labeling was performed via the use of FITC-conjugated antibodies at a concentration of 0.5 mg/mL, which were diluted at a ratio of 1:10 in PBS (pH 7.4). The cells were incubated for 20 min at room temperature in a dark environment, washed with PBS, and centrifuged. All samples were subjected to flow cytometry within 4 h via an FACSCalibur^®^ flow cytometer (Becton Dickinson). Green fluorescence (FITC) was detected in the FL1 channel (530/30 nm), and at least 10,000 events were analyzed on the basis of forward scatter (FSC) and side scatter (SSC) parameters. The data were analyzed via FlowJo^®^ (TreeStar) or WinMDI 2.9 (Joseph Trotter).

At 6 and 21 days of age, one bird per replicate whose weight was around the average of the group (±5%) was euthanized via cervical dislocation following an 8 h fasting period, totaling eight birds per treatment. Histological slides were prepared from 2.0 cm segments of the duodenum, jejunum, and ileum, which were fixed in 10% buffered formalin for 24 h, stored in 70% ethanol, processed according to Luna [22], and stained via the hematoxylin–eosin (H&E) method. Semiserial sections with a thickness of 5 µm were obtained via a rotary microtome (RM2255, Leica Biosystems, Buffalo Grove, IL, USA).

Images were captured at 4× magnification via an optical microscope (E200, Nikon Eclipse, São Paulo, Brazil) connected to a computer and analyzed via Tcapture^®^ software version 3.2. The measurements included villus height, villus width, crypt diameter, and wall thickness, with 20 measurements taken per replicate, totaling 160 per segment per treatment. Villus height was measured from the basal region to the villus apex, villus width was measured from the same villus, crypt diameter was measured from the nearest crypt, and wall thickness was measured from the villus apex to the serosal wall [23].

Histological sections of the duodenum, jejunum, and ileum were further analyzed via the I See Inside^®^ (ISI) scoring system, a numerical index for evaluating histological alterations in the intestine [24]. The assessment included inflammatory cell infiltration, congestion, epithelial desquamation, *Eimeria* oocysts, bacterial clusters, rod-shaped bacteria, cysts, mucus, necrosis, edema, and hemorrhage. Each alteration was assigned an impact factor (IF) on the basis of its effect on organ function, with severity ranging from 1 to 3 [24,25]. The extent of the lesion was scored from 0 to 3, with a score of 0 indicating no alteration, a score of 1 for up to 25% of the affected area, a score of 2 for 25–50%, and a score of 3 for >50%. The final ISI index was calculated as follows:

ISI = Σ (FI × S),

where IF is the impact factor and S is the corresponding lesion score. The total ISI score for each bird was determined by summing all the variables, with each bird considered an experimental unit. A total of 20 villi per intestinal segment per bird were analyzed, resulting in 160 villi per treatment, using the PROPLUS IMAGE 4.1 system (Midia Cybertecnics).

### 2.5. Statistical Analysis

The effects of the treatments (TRs) were analyzed according to the experimental model (Equation):

Yik = μ + (TR)i + eik

where Y represents the response variable, μ is the population mean of the variable, TR represents the effect of treatments, and eik represents the residual error.

The data were analyzed for the presence of outliers (box-and-whisker plot), homogeneity of variances (Bartlett’s test), and normality of residuals (Shapiro–Wilk test). Data with a normal distribution were subjected to analysis of variance (ANOVA), and means were compared via the SNK test using the PROC GLM procedure. This test was performed at a 5% significance level.

For data that did not follow a normal distribution, variables were analyzed using the non-parametric Kruskal–Wallis test at the 5% significance level, which was carried out using the PROC NPAR1WAY procedure. When significant effects (*p* < 0.05) were detected, the means were compared using Dunn’s test. All statistical procedures were conducted using SAS^®^ statistical software [26].

## 3. Results

The use of additives affected the feed intake (*p* < 0.0001), weight gain (*p* < 0.0001), and feed conversion ratio (*p* < 0.0001) of the broilers from 1 to 8 days of age (Table 3). The lowest feed intake was observed in the essential oil treatment group, particularly compared with the treatment group without AGPs and the treatment with the combination of prebiotics 1 and 2. With respect to weight gain and the feed conversion ratio, the birds that received the diet without AGPs presented the worst performance.

The use of additives also affected the feed intake (*p* < 0.0001), weight gain (*p* < 0.0001), and feed conversion ratio (*p* < 0.0001) of the broilers from 9 to 21 days of age (Table 3). The lowest feed intake was observed in the treatment without AGPs, followed by the essential oil treatment. The highest weight gain and best feed conversion ratios were recorded in all the treatments with AGPs compared with those in the treatment without AGPs. Similarly, additives affected the feed intake (*p* < 0.0001), weight gain (*p* < 0.0001), and feed conversion ratio (*p* < 0.0001) of the broilers from 22 to 39 days of age (Table 4).

The lowest feed intake was observed in the birds from the treatment without AGPs compared with those from the other treatments. Compared with those in the treatment without AGPs, the weight gain was greater and the feed conversion ratio was the best in the AGP treatment group. No effect of additive use was observed on broiler performance from 40 to 44 days of age (*p* > 0.05).

For the overall period from 1 to 44 days of age, the use of additives affected the feed intake (*p* < 0.0001), weight gain (*p* < 0.0001), the feed conversion ratio (*p* < 0.0001), viability (*p* < 0.0001), and the productive efficiency index (*p* < 0.0001) (Table 5).

The lowest feed intake, weight gain, and productive efficiency index were observed in the birds from the treatment without AGPs. In terms of the feed conversion ratio, the best results were obtained in the birds fed diets with AGP + zootechnical additives, with the best value recorded for the AGP + essential oil treatment, compared with the treatments without AGPs and with AGPs but without additives. An effect (*p* < 0.0001) was observed for livability; however, the mean comparison test did not reveal significant differences among the treatments.

An effect (*p* < 0.05) was observed on the percentage, occurrence frequency, and phagocytosis index of monocytes in broilers at 6 days of age (Table 6). The highest values for percentage and occurrence frequency were observed in the AGP + essential oil and AGP + butyric acid treatments, whereas the highest phagocytosis index values were recorded in the treatments without AGPs and with AGPs.

In terms of the phagocytic profile of heterophils, a significant effect (*p* < 0.05) was observed for the percentage and occurrence frequency, with the highest values found in the AGP + butyric acid treatment group, with no difference in percentage between the groups receiving AGP + essential oil, AGP + probiotic, and AGP + prebiotic 1 and without AGPs and with no difference in the occurrence frequency compared with the treatment group without AGPs. No effects of the treatments were observed on the total leukocytes (*p* > 0.05) (Table 6).

An effect (*p* < 0.05) was observed for the percentage and occurrence number of phagocytic monocytes at 21 days of age. The highest monocyte percentage was found in the treatment without AGPs, which did not differ statistically from the treatments with AGP and AGP + prebiotics 1 and 2. Similarly, the highest occurrence frequency of phagocytic monocytes was recorded in the treatment without AGPs, differing only from the AGP + prebiotic 1 treatment.

For heterophils at 21 days of age, an effect (*p* < 0.05) was observed for the percentage, occurrence frequency, and phagocytosis index. The highest heterophil percentage was found in the AGP + prebiotics 1 and 2 treatment, which did not differ from the AGP + prebiotic 1 treatment. The highest occurrence frequencies of phagocytic heterophils were observed in the AGP + prebiotics 1 and 2 and AGP + prebiotic 1 treatments, which differed only from the AGP + organic acid treatment, which showed the lowest value. Regarding the phagocytosis index, the highest values were observed in the AGP + prebiotics 1 and 2 and AGP + prebiotic 1 treatments, which differed significantly only from the treatment without AGPs.

No treatment effects were observed on the total leukocyte count (*p* > 0.05) (Table 7).

No effects of the AGPs or zootechnical additives were detected on the total leukocyte count; the percentages of heterophils, lymphocytes, or monocytes; or the heterophil-to-lymphocyte ratio (*p* > 0.05) in birds at 6 and 21 days of age. No effects of the treatments on the birds’ intestinal histomorphometry were observed at 6 days of age (*p* > 0.05). However, an effect was found for the villus height and duodenal wall thickness at 21 days of age (*p* < 0.0001), with a greater villus height and wall thickness observed in the AGP treatment (Table 8).

An effect was observed for the villus height (*p* < 0.0193) and crypt diameter (*p* < 0.0130) in the jejunum at 6 days of age and for the villus height (*p* < 0.0003) and wall thickness (*p* < 0.0085) at 21 days of age. At 6 days, a greater villus height was observed in the birds that received only AGP than in those that received the other treatments, except for the AGP + probiotic group. A greater crypt diameter was found in the birds fed diets without AGPs, with AGP + prebiotics 1 and 2, with AGP + organic acid, and with AGP + essential oil than in the birds fed only AGP or AGP + prebiotic 1. At 21 days of age, a greater villus height was observed in the birds fed AGP diets than in the birds that did not receive AGPs or those fed AGP + probiotics.

Additionally, a greater wall thickness at 21 days was observed in the birds fed AGP diets than in those that did not receive AGP or in the birds fed AGP + probiotic or AGP + essential oil (Table 9). An effect was observed for the villus height (*p* < 0.0008) and wall thickness (*p* < 0.0058) in the ileum at 6 days of age, as well as for the villus height (*p* < 0.0083) and wall thickness (*p* < 0.0138) at 21 days of age. At 6 days, a greater villus height was observed in the birds fed AGP diets.

A greater wall thickness was recorded in the birds fed AGP than in the birds fed AGP + prebiotics 1 and 2. At 21 days of age, a greater villus height was observed in the birds that received AGP than in those that received the other treatments, except for the birds that were fed AGP + organic acid. A greater wall thickness was observed in the birds fed AGP and AGP + prebiotics 1 and 2, which did not differ from the other treatments, except for the birds fed AGP + organic acids (Table 10).

An effect was observed for inflammatory infiltration (*p* < 0.0001), congestion (*p* < 0.0001), desquamation (*p* < 0.0001), cysts (*p* < 0.0115), edema (*p* < 0.0001), and hemorrhage (*p* < 0.0197) in the duodenum of broilers at 6 days of age. Reduced inflammatory infiltration was recorded in the birds fed diets with AGP and AGP + essential oil. The lowest level of congestion was observed in the birds fed AGP, which did not differ from those receiving AGP + prebiotics 1 and 2 or AGP + essential oil.

Desquamation was lowest in the birds receiving only AGP. A lower degree of crypt cyst formation was observed in the birds receiving treatments without AGPs, with AGP + prebiotic 1, and with AGP + essential oil than in the birds receiving AGP and AGP + probiotic. A lower level of edema was recorded in the birds fed AGP than in those fed diets without AGPs, AGP + essential oil, or AGP + organic acid. Hemorrhaging was reduced in all the treatment groups except for the AGP + organic acid group (Table 11).

At 21 days, the inside index for the duodenum had effects on inflammatory infiltration (*p* < 0.0001) and desquamation (*p* < 0.0100). Reduced inflammatory infiltration was observed in the birds receiving diets with AGP, AGP + probiotic, AGP + essential oil, or AGP + organic acid. The lowest desquamation levels were recorded in the duodenum of the birds fed AGP (Table 11).

For the inside index in the jejunum at 6 days of age, an effect was observed for inflammatory infiltration (*p* < 0.0001), congestion (*p* < 0.0001), desquamation (*p* < 0.0001), necrosis (*p* < 0.0001), and edema (*p* < 0.0058). Reduced inflammatory infiltration was found in the birds fed diets with AGP, AGP + essential oil, or AGP + organic acid. Congestion was lowest in the birds fed AGP + prebiotics 1 and 2, without differing from those receiving AGP and AGP + prebiotic 1.

A lower degree of desquamation was observed in the birds fed AGP, which did not differ from those receiving AGP + prebiotics 1 and 2, AGP + prebiotic 1, or AGP + essential oil. Less edema in the jejunum was recorded in the birds fed AGP, AGP + prebiotics 1 and 2, AGP + prebiotic 1, and AGP + probiotic (Table 12).

At 21 days of age, the inside index for the jejunum had effects only on inflammatory infiltration (*p* < 0.0003), with all the treatments reducing this parameter except for the birds that did not receive AGPs (Table 12).

For the inside index in the ileum at 6 days of age, an effect was observed for inflammatory infiltration (*p* < 0.0001), congestion (*p* < 0.0013), desquamation (*p* < 0.0001), necrosis (*p* < 0.0001), and edema (*p* < 0.0103). Reduced inflammatory infiltration was found in the birds fed AGP diets. The lowest congestion values were observed in the birds fed AGP, which did not differ from those receiving AGP + prebiotics 1 and 2 and AGP + prebiotic 1.

A lower degree of desquamation was recorded in the birds fed AGP and AGP + prebiotics 1 and 2 than in those receiving AGP + prebiotic 1 or AGP + essential oil. The treatments with AGP reduced necrosis in the ileum, regardless of the addition of zootechnical additives. The lowest edema values were observed in the birds fed AGP + prebiotic 1, which did not differ from the other treatments except for the group receiving AGP + organic acid (Table 13).

At 21 days of age, an effect on inflammatory infiltration (*p* < 0.0020), congestion (*p* < 0.0028), desquamation (*p* < 0.0274), necrosis (*p* < 0.0226), and edema (*p* < 0.0005) in the ileum was detected. The treatments reduced inflammatory infiltration and necrosis in the ileum, except when the birds did not receive AGPs. A lower level of congestion was recorded in the birds fed AGP than in those not fed AGP, AGP + prebiotics 1 and 2, AGP + prebiotic 1, and AGP + essential oil. Desquamation was reduced in all the treatments except for the birds receiving AGP + organic acid. Necrosis was also reduced by the various treatments, except in the birds fed AGP + essential oil (Table 13).

## 4. Discussion

An analysis of the broiler performance results indicated that the use of AGPs provided benefits in terms of weight gain, feed intake, the feed conversion ratio, and the productive efficiency index across all studied phases, regardless of whether they were used alone or in combination with zootechnical additives. However, over the entire period (1 to 44 days of age), the feed conversion ratio was greater in the treatments in which AGPs were combined with zootechnical additives, with a reduction of 0.09 points observed in the AGP + essential oil treatment compared to the AGP treatment without additives.

Recent studies have focused on the importance of zootechnical additives as modulators of the intestinal microbiota in broilers, hypothesizing that this effect enhances intestinal absorption, increases digestive enzyme secretion, reduces nitrogen excretion, and neutralizes enterotoxins, leading to significant improvements in both performance and bird welfare [27,28].

Moraes et al. [29] reported that the inclusion of castor bean and cashew nutshell extracts (0.15%) in broiler diets from 1 to 28 days of age improved bird performance, yielding results comparable to those obtained with monensin. This effect was attributed to the antimicrobial properties of these additives, which act as intestinal microbiota modulators, particularly against Gram-positive bacteria such as Clostridium perfringens and Staphylococcus aureus.

In animal nutrition, two key mechanisms highlight the potential of these additives: the stimulation of endogenous enzyme production and regulation of the intestinal microbiota. Both contribute to maintaining poultry health and performance, as essential oils prevent pathogenic bacteria from colonizing the intestinal mucosa [30]. In a study using essential oils, Bess et al. [31] reported improvements in broiler performance (weight gain and the feed conversion ratio), attributing these benefits to the antimicrobial and anti-inflammatory activities of essential oils.

Since the 1940s, AGPs have been widely used to increase the immunocompetence of birds against diseases and as growth promoters. However, their prophylactic use can contribute to the development of drug-resistant bacteria [32]. Therefore, studies evaluating the effects of zootechnical additives, both alone and in combination with AGPs, are crucial for developing efficient nutritional strategies aimed at reducing the use of AGPs in broiler nutrition [3,10,30].

At 6 days of age, the phagocytic profile analysis revealed a greater percentage of monocytes in the groups treated with AGP + essential oil and AGP + butyric acid. However, these treatments resulted in a lower phagocytosis index for monocytes, likely due to the mechanism of action of these additives, reinforcing their immunomodulatory capacity when combined with AGP. Zootechnical additives can be used as tools to enhance acquired immune responses in poultry, thereby improving performance by reducing challenges from infectious agents [33].

Moraes et al. [34] reported that the inclusion of castor bean and cashew nut shell extracts (0.15%) in broiler diets increased the expression of interferon (IFN), interleukin 6 (IL-6), and tumor necrosis factor (TNF), whereas the control group presented an increased expression of cyclooxygenase (COX) and interleukin 1 (IL-1). This modulated the inflammatory response against *Eimeria* spp., leading the authors to conclude that the evaluated essential oils were effective tools for modulating the immune system in birds affected by coccidiosis [34].

In addition to essential oil and butyric acid combined with AGP, treatments with prebiotics or probiotics also resulted in a lower phagocytosis index at 6 days of age. Prebiotics promote the growth of two lactic-acid-producing bacteria (*Bifidobacterium* and *Lactobacillus*), which are beneficial for intestinal health. The proliferation of these bacteria aids in the production of bacteriocins, which inhibit the development of pathogenic bacteria in the gastrointestinal tract of poultry [35]. This mechanism may explain the lower phagocytosis index observed in birds fed prebiotics.

At 22 days of age, the birds receiving additives + AGP or AGP alone presented a lower percentage of monocytes, although the phagocytosis index remained unaffected. For heterophils, a greater percentage was observed in the treatments with one or two prebiotics; however, the highest heterophil activation (phagocytosis index) occurred in all the treatments with AGP, regardless of the presence of zootechnical additives. Thus, in the initial phase, the positive effects of additives on phagocytic activity were more evident, even in the absence of AGPs, a trend that was not observed during the grower phase. The obtained values indicate that the birds were in good health, as they were within the normal reference ranges.

The results of this study demonstrate that the association of additives with AGP is more relevant during the initial phase than during the grower phase. This finding is supported by Song et al. [36], who evaluated the development of the immune system in broilers according to age and reported that immunological indicators continue to increase until 34 days of age. These findings suggest that during this period, the immune system of broilers is still maturing.

Moreover, epidemiological studies have provided evidence that immune system development is closely linked to the role of the intestinal microbiota [37]. The early use of AGP can negatively affect this microbiota, potentially impairing immune system function later in life [37]. Therefore, the immune system can be adjusted during the early grower phase through immunomodulatory nutrition strategies that incorporate zootechnical additives, promoting rapid immune development and maturation in broilers.

With respect to the intestinal histomorphometry and inside index evaluations, the results indicate that the use of zootechnical additives combined with AGP led to improvements. However, these effects were not superior to those observed in the birds fed AGP alone. These findings suggest that the dosage and supplementation period of zootechnical additives should be optimized to maximize their effects on the intestinal health of broilers. Depending on the dosage, additives may have either beneficial or detrimental effects on animals [38].

In a study conducted by Stefanello et al. [39], the authors concluded that broilers fed diets containing AGPs presented improved performance, digestibility, and intestinal integrity compared with those of birds not receiving AGPs. The beneficial effects of AGPs on broiler performance and intestinal health are well documented in the literature [40], likely due to their role in reducing the severity of intestinal diseases, stabilizing microbial populations, lowering inflammatory responses, and suppressing bacterial pathogens in the gastrointestinal tract [40,41]. However, with increasing restrictions on AGP use in poultry production, concerns regarding the incidence of intestinal diseases are increasing. As a result, investing in research in this field is essential for developing strategies to address this ongoing challenge for the poultry industry.

The I See Inside (ISI) methodology, according to Belote et al. [25], indicates that inflammatory parameters can evolve even in the absence of specific pathogens, emphasizing the influence of diet and environmental factors. In uncontrolled environments, variations in these parameters may occur, suggesting that maintaining gastrointestinal tract balance is crucial for pathogen control, microbiota stability, and reducing the incidence of enteric diseases [42].

The results of this study highlight the importance of zootechnical additives as complementary tools to AGPs in broiler production, especially in enhancing immune function and optimizing feed conversion. While AGPs continue to be effective in promoting intestinal health and performance, the strategic use of additives can further support gut microbiota balance and immune modulation. The growing restrictions on antimicrobial use in poultry production reinforce the need for continued research to develop alternative strategies that maintain bird performance and health while reducing reliance on antibiotics.

## 5. Conclusions

Zootechnical additives, when combined with antibiotic growth promoters (AGPs), improve the feed conversion efficiency in broilers from 1 to 44 days of age and play a key role in supporting immune system development, particularly during the early grower phase. Essential oils were especially effective in promoting both performance and immune response. However, their effects on intestinal integrity were not superior to those of AGPs alone. These findings highlight the potential of zootechnical additives as complementary tools in poultry production and reinforce the need for further studies to optimize their use and reduce antibiotic dependence.

## Figures and Tables

**Table 1:** Description of the experimental treatments.

Treatments	Inclusion g/100 kg of Feed and Stages Used
Without AGP+	Total
With AGP	Total
With AGP + 1 * & 2 ** prebiotics	Prebiotic 1 *—20 g pre-starter and 10 g starter
	Prebiotic 2 **—10 g grower and finisher
With AGP + 1 * prebiotic	20 g pre-starter, 10 g starter, grower, and finisher
With AGP + probiotic 1	1 g total
With AGP + essential oil 2	7.5 g total
With AGP + butyric acid 3	10 g pre-starter and starter; 7.5 g grower, and 5 g finisher

+AGP: Antibiotic growth promoters. * Prebiotic with the yeast cell wall, yeast extract, fructosaccharides, galactooligosaccharides, copper, magnesium, manganese and zinc amino acid chelate, selenium proteinate, and dry sugarcane yeast. ** Prebiotic composed of *Saccharomyces cerevisiae* hydrolyzed by enzymes, yeast wall and extract, 1.3 and 1.6 beta-glucans, and mannan oligosaccharides. 1: Probiotic with a defined strain (*Bacillus subtilis* LFU160) at 1.0 × 10^8^ CFU/g, dry sugarcane yeast at 87.66%, silicone dioxide, and skim milk powder. 2: Essential oils derived from cashew nut shell liquid (CNSL), cardol, cardanol, and ribonucleic acid. 3: Organic acid with glycerides of butyric acid, silica, free fatty acid in butyric acid, and free glycerol.

**Table 2:** Calculated proximate and nutritional compositions of the experimental diets.

Ingredients	Pre-Starter	Initial	Grower	Finisher
Corn grain	57.77	59.52	59.77	66.93
Soybean meal 45%	34.30	32.10	31.70	25.00
Meat meal 45%	4.45	3.90	2.50	2.45
Limestone 38%	0.49	0.51	0.52	0.53
Poultry fat	1.18	2.20	3.93	3.49
Common white salt	0.37	0.37	0.34	0.31
Sodium bicarbonate	0.10	0.10	0.10	0.10
DL-methionine 99%	0.02	0.00	0.05	0.05
L-lysine 78%	0.05	0.00	0.00	0.03
Threonine 98%	0.02	0.00	0.00	0.00
Mycotoxin adsorbent	0.10	0.10	0.00	0.00
Penta copper sulphate (25%)	0.05	0.05	0.05	0.05
Emulsifier	0.00	0.05	0.05	0.05
Vitamin, mineral, and enzyme supplement 1	1.10	1.10	0.00	0.00
Vitamin, mineral, and enzyme supplement 2	0.00	0.00	1.00	0.00
Vitamin, mineral, and enzyme supplement 3	0.00	0.00	0.00	1.00
Total (kg)	100.00	100.00	100.00	100.00
**Calculated Nutritional Composition**				
Metabolizable energy (Kcal/kg)	3.050	3.150	3.270	3.300
Gross protein (%)	23.75	22.52	21.65	18.96
Crude fiber (%)	2.39	2.32	2.34	2.16
Crude fat (%)	4.23	5.21	6.76	6.49
Ash (%)	5.78	5.47	4.96	4.58
Moisture (%)	11.83	11.77	11.68	11.78
Calcium (%)	1.01	0.95	0.80	0.78
Total phosphorus (%)	0.59	0.55	0.47	0.44
Total sodium (%)	0.22	0.22	0.20	0.19
Total sulfur amino acids (%)	0.19	0.19	0.15	0.17
Lysine (%)	1.44	1.44	1.21	1.30

1. Vitamin, mineral, and enzymatic supplement composition (per kg of feed) of pre-starter and starter: BHT 0.1 g/kg; Phytase 1000.0 U/kg; Glucanase 250.0 TGU; Narasin 50.0 mg/kg; Nicarbazine 50.0 mg/kg; Protease 2000 U/kg; Xylanase 560 TXU; Lysine 1.8 g/kg; Methionine 3.2 g/kg; Threonine 0.6 g/kg; Enramycin 10.0 mg/kg; Folic Acid 1.4 mg/kg; Pantothenic Acid 13.6 mg/kg; Biotin 0.1 mg/kg; Copper 12.0 mg/kg; Choline 0.3 g/kg; Iron 50.0 mg/kg; Iodine 1.5 mg/kg; Manganese 8.0 mg/kg; Niacin 39.0 mg/kg; Selenium 0.3 mg/kg; Vitamin An 8000.0 IU/kg; Vitamin B1 2.0 mg/kg; Vitamin B12 14.8 mcg/kg; Vitamin B2 5.8 mg/kg; Vitamin B2. 2. Vitamin, mineral, and enzyme supplement composition (per kg of feed) of grower: BHT 0.1 g/kg; Phytase 1000.0 U/kg; Glucanase 250.0 TGU; Protease 2000.0 U/kg; Salomycin 66.0 mg/kg; Xylanase 560.0TXU; Lysine 1.5 g/kg; Methionine 2.5 g/kg; Threonine 0.4 g/kg; Enramycin 10.0 mg/kg; Folic Acid 1.2 mg/kg; Pantothenic Acid 12.0 mg/kg; Biotin 0.1 mg/kg; Copper 12.0 mg/kg; Choline 0.3 g/kg; Iron 50.0 mg/kg; Iodine 1.5 mg/kg; Manganese 80.0 mg/kg; Niacin 33.0 mg/kg; Selenium 0.3 mg/kg; Vitamin A 6500.0 IU/kg; Vitamin B 1.6 mg/kg; Vitamin B12 12.1 mcg/kg; Vitamin B2 5.0 mg/kg; Vitamin B6 2.4 mg/kg; Vitamin Delen. 3 Vitamin, mineral, and enzyme supplement composition (per kg of feed) of finisher-BHT 0.1 g/kg; Phytase 1000.0 U/kg; Glucanase 250.0 TGU; Xylanase 560.0 TXU; Lysine 1.8 g/kg; Methionine 2.4 g/kg; Threonine 0.6 g/kg; Enramycin 5.0 mg/kg; Pantothenic Acid 7.1 mg/kg; Copper 12.0 mg/kg; Choline 0.4 g/kg; Iron 50.0 mg/kg; Iodine 1.5 mg/kg; Manganese 80.0 mg/kg; Niacin 20.4 mg/kg; Selenium 0.2 mg/kg; Vitamin A 1965.0 IU/kg; Vitamin B 4.7 mcg/kg; Vitamin B2 2.4 mg/kg; Vitamin D3 550.0 IU/kg; Vitamin E 5.5 IU/kg; Vitamin K 30.6 mg/kg; Zinc 80.0 mg/kg.

**Table 3:** Effects of treatments on the performance of broilers from 1 to 8 days of age and from 9 to 21 days of age.

Treatments	Feed Intake (g)	Weight Gain (g)	Feed Conversion Ratio (g/g)	Feed Intake (g)	Weight Gain (g)	Feed Conversion (g/g)
	1 to 8 Days Old			9 to 21 Days of Age		
Without AGPs	169.07 ± 1.69 ^a^	117.92 ± 1.80 ^b^	1.41 ± 0.02 ^a^	713.00 ± 8.92 ^c^	395.02 ± 8.64 ^b^	1.80 ± 0.02 ^a^
With AGP	148.05 ± 2.79 ^bc^	154.21 ± 1.23 ^a^	0.96 ± 0.02 ^b^	882.41 ± 25.51 ^a^	538.73 ± 20.10 ^a^	1.64 ± 0.04 ^b^
AGP + prebiotics 1 and 2	156.12 ± 2.38 ^b^	154.56 ± 3.65 ^a^	1.01 ± 0.01 ^b^	878.61 ± 20.65 ^a^	547.69 ± 21.24 ^a^	1.61 ± 0.02 ^b^
AGP + prebiotic 1	152.98 ± 1.51 ^bc^	149.03 ± 3.15 ^a^	1.01 ± 0.02 ^b^	854.21 ± 14.59 ^a^	527.70 ± 12.82 ^a^	1.62 ± 0.01 ^b^
AGP + probiotic	156.12 ± 2.04 ^b^	153.45 ± 2.71 ^a^	1.01 ± 0.09 ^b^	890.73 ± 21.20 ^a^	555.20 ± 17.69 ^a^	1.60 ± 0.02 ^b^
AGP + essential oil	146.74 ± 1.81 ^c^	155.20 ± 3.17 ^a^	0.94 ± 0.02 ^b^	799.30 ± 9.51 ^b^	525.41 ± 10.54 ^a^	1.52 ± 0.03 ^b^
AGP + organic acid	153.51 ± 1.37 ^bc^	154.71 ± 1.17 ^a^	0.99 ± 0.009 ^b^	888.15 ± 22.11 ^a^	554.87 ± 26.11 ^a^	1.61 ± 0.038 ^b^
Probability	<0.0001	<0.0001	<0.0001	<0.0001	<0.0001	<0.0001
CV (%)	3.65	4.93	4.93	6.20	9.63	5.52

±Standard error. a-b-c: Means followed by different letters are distinguished by the SNK test at 5% probability level. AGP: Antibiotic growth promoters. Prebiotic 1: prebiotics with a yeast cell wall, yeast extract, fructooligosaccharides, and galactooligosaccharides. Prebiotic 2: prebiotic composed of enzyme-hydrolyzed *Saccharomyces cerevisiae*, yeast wall and extract, 1.3 and 1.6 beta glucans, and mannan oligosaccharides. CV (%): coefficient of variation.

**Table 4:** Effects of treatments on the performance of broilers from 22 to 39 days of age.

Treatments	Feed Intake (g)	Weight Gain (g)	Feed Conversion (g/g)
	22 to 39 Days of Age		
Without AGPs	2473 ± 46.46 ^b^	1301 ± 38.01 ^b^	1.901 ± 0.02 ^a^
With AGP	2975 ± 31.97 ^a^	1799 ± 20.22 ^a^	1.653 ± 0.01 ^b^
AGP + prebiotics 1 and 2	2895 ± 18.93 ^a^	1761 ± 11.83 ^a^	1.642 ± 0.01 ^b^
AGP + prebiotic 1	2947 ± 38.23 ^a^	1768 ± 47.26 ^a^	1.673 ± 0.03 ^b^
AGP + probiotic	2948 ± 36.53 ^a^	1777 ± 35.51 ^a^	1.661 ± 0.02 ^b^
AGP + essential oil	2969 ± 26.14 ^a^	1814 ± 20.91 ^a^	1.630 ± 0.01 ^b^
AGP + organic acid	2980 ± 41.17 ^a^	1824 ± 22.99 ^a^	1.632 ± 0.03 ^b^
Probability	<0.0001	<0.0001	<0.0001
CV (%)	3.46	4.99	4.30

±Standard error. a-b: Means followed by different letters in the columns differ significantly according to the SNK test at the 5% probability level. AGP: Antibiotic growth promoters. Prebiotic 1: prebiotics with a yeast cell wall, yeast extract, fructooligosaccharides, and galactooligosaccharides. Prebiotic 2: prebiotic composed of *Saccharomyces cerevisiae* hydrolyzed by enzymes, yeast wall and extract, 1.3 and 1.6 beta glucans, and mannan oligosaccharides. CV (%): coefficient of variation.

**Table 5:** Effects of various treatments on the performance of broilers aged 1 to 44 days.

Treatments	Feed Intake (g)	Weight Gain (kg)	Feed Conversion (g/g)	Livability (%)	Productive Efficiency Index
	1 to 44 Days of Age				
Without AGPs	4323 ± 66.54 ^b^	2324 ± 49.44 ^b^	1.862 ± 0.014 ^a^	94.00 ± 1.51 ^b^	267.55 ± 10.02 ^b^
With AGP	4893 ± 53.09 ^a^	2949 ± 18.85 ^a^	1.661 ± 0.010 ^b^	99.00 ± 0.65 ^a^	400.10 ± 3.23 ^a^
AGP + prebiotics 1 and 2	4877 ± 51.01 ^a^	2992 ± 38.00 ^a^	1.630 ± 0.009 ^bc^	97.71 ± 1.18 ^a^	400.05 ± 7.72 ^a^
AGP + prebiotic 1	4907 ± 46.73 ^a^	2986 ± 36.02 ^a^	1.643 ± 0.008 ^bc^	94.50 ± 1.9 ^b^	390.58 ± 9.24 ^a^
AGP + probiotic	4954 ± 53.09 ^a^	3013 ± 38.33 ^a^	1.640 ± 0.024 ^bc^	96.57 ± 1.61 ^ab^	381.89 ± 11.69 ^a^
AGP + essential oil	4848 ± 46.92 ^a^	3.017.23 ± 28.77 ^a^	1.572 ± 0.022 ^c^	94.95 ± 1.26 ^b^	420.52 ± 11.41 ^a^
AGP + organic acid	5001 ± 38.15 ^a^	3.060.55 ± 37.90 ^a^	1.63 ± 0.024 ^bc^	94.00 ± 0.75 ^b^	400.46 ± 9.70 ^a^
Probability	<0.0001	<0.0001	<0.0001	<0.0001	<0.0001
CV (%)	3.01	3.54	3.02	3.53	6.86

±Standard error. a-b-c: Averages followed by different letters in the columns are distinguished by the SNK test at 5% probability level. AGP: Antibiotic growth promoters. Prebiotic 1: prebiotics with a yeast cell wall, yeast extract, fructooligosaccharides, and galactooligosaccharides. Prebiotic 2: prebiotic composed of enzyme-hydrolyzed *Saccharomyces cerevisiae*, yeast wall and extract, 1.3 and 1.6 beta glucans, and mannan oligosaccharides. CV (%): coefficient of variation.

**Table 6:** Evaluation of the phagocytic effects on the total number of leukocytes, percentage (%), number of occurrences (N), and phagocytosis index (PI *) of monocytes and heterophiles of broilers at 6 days of age.

Treatments	Total Leukocytes	Phagocytic Monocytes			Phagocytic Heterophile		
		(%)	Number of Occurrences	Phagocytosis Index	(%)	Occurrence Number	Phagocytic Index
Without AGPs	40.8	1.6 ^c^	0.6 ^c^	664.5 ^a^	6.4 ^ab^	2.7 ^ab^	416.7
With AGP	41.0	2.0 ^c^	0.8 ^c^	620.9 ^ab^	4.0 ^b^	1.7 ^b^	543.7
AGP + prebiotics 1 and 2	40.2	5.7 ^bc^	2.5 ^bc^	396.8 ^bc^	4.3 ^b^	1.7 ^b^	412.0
AGP + prebiotic 1	42.1	7.1 ^b^	3.0 ^b^	352.2 ^c^	9.1 ^ab^	3.8 ^b^	417.8
AGP + probiotic	41.9	6.0 ^b^	2.5 ^b^	388.7 ^bc^	5.5 ^ab^	2.3 ^b^	359.5
AGP + essential oil	41.5	13.9 ^a^	5.8 ^a^	233.3 ^c^	8.2 ^ab^	3.4 ^b^	435.9
AGP + organic acid	43.8	16.0 ^a^	7.5 ^a^	268.9 ^c^	10.7 ^a^	4.8 ^a^	291.9
Probability	0.2395	<0.0001	<0.0001	0.0013	0.0014	0.0173	0.2372

a-b-c: Means followed by different letters in the columns are distinguished by the Kruskal–Wallis test at 5% probability. AGP: Antibiotic growth promoters. Prebiotic 1: prebiotics with a yeast cell wall, yeast extract, fructooligosaccharides, and galactooligosaccharides. Prebiotic 2: prebiotic composed of enzyme-hydrolyzed *Saccharomyces cerevisiae*, yeast wall and extract, 1.3 and 1.6 beta glucans, and mannan oligosaccharides. CV (%): coefficient of variation.

**Table 7:** Evaluation of the phagocytic effects on the total number of leukocytes, percentages (%), number of occurrences (N), and phagocytosis index (PI *) of monocytes and heterophiles in 21-day-old broilers.

Treatments	Total Leukocytes	Phagocytic Monocytes			Phagocytic Heterophile		
		(%)	Occurrence Number	Phagocytosis Index	(%)	Occurrence Number	Phagocytosis Index
Without AGPs	29,636.2	0.8 ^a^	187.9 ^a^	237.7	1.8 ^bc^	412.2 ^ab^	250.5 ^b^
With AGP	22,809.4	0.7 ^ab^	152.6 ^ab^	321.3	1.9 ^bc^	464.9 ^ab^	291.4 ^ab^
AGP + prebiotics 1 and 2	20,200.7	0.6 ^abc^	123.2 ^b^	402.7	3.2 ^a^	564.1 ^a^	442.6 ^a^
AGP + prebiotic 1	20,951.0	0.5 ^bc^	101.0 ^b^	446.4	2.8 ^ab^	529.0 ^a^	400.1 ^a^
AGP + probiotic	26,675.3	0.5 ^bc^	121.5 ^b^	397.7	1.9 ^bc^	383.7 ^ab^	397.0 ^ab^
AGP + essential oil	23,361.8	0.4 ^c^	98.2 ^b^	387.5	1.5 ^c^	338.2 ^ab^	319.2 ^ab^
AGP + organic acid	17,207.5	0.6 ^bc^	95.4 ^b^	430.5	1.7 ^bc^	285.7 ^b^	273.7 ^ab^
Probability	0.1132	0.0008	0.0048	0.0881	0.0004	0.0097	0.0048

a-b-c: Means followed by different letters in the columns are distinguished by the Kruskal–Wallis test at 5% probability level. AGP: Antibiotic growth promoters. Prebiotic 1: prebiotics with a yeast cell wall, yeast extract, fructooligosaccharides, and galactooligosaccharides. Prebiotic 2: prebiotic composed of enzyme-hydrolyzed *Saccharomyces cerevisiae*, yeast wall and extract, 1.3 and 1.6 beta glucans, and mannan oligosaccharides.

**Table 8:** Intestinal histomorphometry of the duodenum of broilers according to treatment.

Treatments	Villus Height	Villus Width	Crypt Depth	Total Wall Thickness
	µm			
Without AGPs	1.15 ± 0.08 ^b^	0.20 ± 0.01	0.10 ± 0.01	1.54 ± 0.07 ^b^
With AGP	1.82 ± 0.12 ^a^	0.14 ± 0.02	0.09 ± 0.02	2.21 ± 0.12 ^a^
AGP + prebiotics 1 and 2	1.20 ± 0.09 ^b^	0.22 ± 0.02	0.12 ± 0.02	1.60 ± 0.09 ^b^
AGP + prebiotic 1	1.02 ± 0.13 ^b^	0.23 ± 0.01	0.11 ± 0.01	1.40 ± 0.13 ^b^
AGP + probiotic	1.05 ± 0.08 ^b^	0.21 ± 0.02	0.11 ± 0.02	1.53 ± 0.09 ^b^
AGP + essential oil	0.91 ± 0.19 ^b^	0.21 ± 0.03	0.10 ± 0.01	1.32 ± 0.20 ^b^
AGP + organic acid	0.83 ± 0.07 ^b^	0.21 ± 0.03	0.11 ± 0.01	1.30 ± 0.09 ^b^
Probability	0.0001	0.1734	0.3601	0.0001
CV (%)	28.87	30.24	30.34	21.32

± Standard error. AGP: Antibiotic growth promoters. a-b:Different means of treatment with AGP according to Dunnett’s test. Prebiotic 1: prebiotics with a yeast cell wall, yeast extract, fructooligosaccharides, and galactooligosaccharides. Prebiotic 2: prebiotic composed of *Saccharomyces cerevisiae* hydrolyzed by enzymes, yeast wall and extract, 1.3 and 1.6 beta glucans, and mannan oligosaccharides. CV (%): coefficient of variation.

**Table 9:** Intestinal histomorphometry of the jejunum of broilers according to treatment.

Treatments	6-Day-Old		21-Day-Old	
	Villus Height	Crypt Depth	Villus Height	Total Wall Thickness
	µm		µm	
Without AGPs	0.58 ± 0.11 ^ab^	0.11 ± 0.01 ^a^	1.05 ± 0.09 ^ab^	1.46 ± 0.09 ^ab^
With AGP	0.97 ± 0.07 ^a^	0.09 ± 0.01 ^ab^	1.31 ± 0.04 ^a^	1.71 ± 0.08 ^a^
AGP + prebiotics 1 and 2	0.67 ± 0.04 ^ab^	0.11 ± 0.01 ^a^	0.71 ± 0.05 ^b^	1.13 ± 0.07 ^b^
AGP + prebiotic 1	0.72 ± 0.11 ^ab^	0.08 ± 0.01 ^ab^	0.86 ± 0.07 ^b^	1.26 ± 0.10 ^b^
AGP + probiotic	0.53 ± 0.04 ^b^	0.07 ± 0.01 ^b^	1.00 ± 0.16 ^ab^	1.43 ± 0.18 ^ab^
AGP + essential oil	0.65 ± 0.09 ^ab^	0.11 ± 0.01 ^a^	0.92 ± 0.10 ^b^	1.37 ± 0.10 ^ab^
AGP + organic acid	0.64 ± 0.09 ^ab^	0.11 ± 0.01 ^a^	0.70 ± 0.08 ^b^	1.18 ± 0.10 ^b^
Probability	0.0193	0.0130	0.0003	0.0085
CV (%)	33.98	28.70	27.97	23.66

± Standard error. AGP: Antibiotic growth promoters. Different means of treatment with AGP according to Dunnett’s test. a-b: means followed by letters differ statistically by Tukey’s test. Prebiotic 1: prebiotics with a yeast cell wall, yeast extract, fructooligosaccharides, and galactooligosaccharides. Prebiotic 2: prebiotic composed of enzyme-hydrolyzed *Saccharomyces cerevisiae*, yeast wall and extract, 1.3 and 1.6 beta glucans, and mannan oligosaccharides. CV (%): coefficient of variation.

**Table 10:** Intestinal histomorphometry of the ileum of broilers according to treatment.

Treatments	6-Day-Old		21-Day-Old	
	Villus Height	Total Wall Thickness	Villus Height	Total Wall Thickness
	µm		µm	
Without AGPs	0.47 ± 0.06 ^b^	0.84 ± 0.08 ^b^	0.76 ± 0.09 ^ab^	1.14 ± 0.11 ^ab^
With AGP	0.86 a ± 0.06 ^a^	1.26 ± 0.06 ^a^	0.93 ± 0.06 ^a^	1.34 ± 0.08 ^a^
AGP + prebiotics 1 and 2	0.58 ± 0.05 ^b^	1.11 ± 0.07 ^ab^	0.84 ± 0.05 ^ab^	1.40 ± 0.07 ^a^
AGP + prebiotic 1	0.58 ± 0.08 ^b^	1.00 ± 0.08 ^b^	0.69 ± 0.06 ^ab^	1.19 ± 0.09 ^ab^
AGP + probiotic	0.53 ± 0.04 ^b^	0.91 ± 0.05 ^b^	0.68 ± 0.07 ^ab^	1.13 ± 0.09 ^ab^
AGP + essential oil	0.55 ± 0.05 ^b^	0.97 ± 0.08 ^b^	0.72 ± 0.04 ^ab^	1.17 ± 0.06 ^ab^
AGP + organic acid	0.50 ± 0.07 ^b^	0.92 ± 0.07 ^b^	0.59 ± 0.04 ^b^	0.98 ± 0.05 ^b^
Probability	0.0008	0.0058	0.0083	0.0138
CV (%)	29.16	20.03	23.66	18.72

± Standard error. AGP: Antibiotic growth promoters. a-b: means followed by different letters differ statistically according to the SNK test. Prebiotic 1: prebiotics with a yeast cell wall, yeast extract, fructooligosaccharides, and galactooligosaccharides. Prebiotic 2: prebiotic composed of enzyme-hydrolyzed *Saccharomyces cerevisiae*, yeast wall and extract, 1.3 and 1.6 beta glucans, and mannan oligosaccharides. CV (%): coefficient of variation.

**Table 11:** Indexes are shown inside the duodenum of broilers according to treatment at 6 and 21 days of age.

Treatments	6-Day-Old						21-Day-Old	
	Inflammatory Infiltrate	Congestion	Desquamation	Cysts	Edema	Hemorrhage	Inflammatory Infiltrate	Desquamation
	(%)						(%)	
Without AGPs	1.13 ^a^	2.13 ^a^	2.13 ^a^	0.00 ^b^	0.25 ^bc^	0.13 ^b^	1.00 ^a^	1.50 ^a^
With AGP	0.13 ^b^	1.13 ^b^	0.13 ^c^	0.25 ^ab^	0.00 ^c^	0.00 ^b^	0.13 ^b^	0.25 ^b^
AGP + prebiotics 1 and 2	1.50 ^a^	1.75 ^ab^	1.13 ^ab^	0.75 ^a^	0.75 ^ab^	0.00 ^b^	0.88 ^a^	1.88 ^a^
AGP + prebiotic 1	1.00 ^a^	2.13 ^a^	1.38 ^ab^	0.00 ^b^	1.00 ^a^	0.00 ^b^	1.13 ^a^	1.75 ^a^
AGP + probiotic	1.00 ^a^	2.13 ^a^	1.75 ^ab^	0.50 ^ab^	0.88 ^a^	0.00 ^b^	0.13 ^b^	1.88 ^a^
AGP + essential oil	0.13 ^b^	1.75 ^ab^	0.88 ^b^	0.00 ^b^	0.50 ^abc^	0.00 ^b^	0.00 ^b^	1.88 ^a^
AGP + organic acid	1.13 ^a^	2.38 ^a^	2.00 ^a^	0.25 ^ab^	0.25 ^bc^	0.50 ^a^	0.25 ^b^	2.25 ^a^
Probability	0.0001	0.0001	0.0001	0.0115	0.0001	0.0197	0.0001	0.0100

AGP: Antibiotic growth promoters. a-b-c: means followed by different letters in the columns are distinguished by the Kruskal–Wallis test at 5% probability level. Prebiotic 1: prebiotics with a yeast cell wall, yeast extract, fructooligosaccharides, and galactooligosaccharides. Prebiotic 2: prebiotic composed of enzyme-hydrolyzed *Saccharomyces cerevisiae*, yeast wall and extract, 1.3 and 1.6 beta glucans, and mannan oligosaccharides.

**Table 12:** Indexes are shown inside the jejunum of broilers according to treatment at 6 and 21 days of age.

Treatments	6-Day-Old					21-Day-Old
	Inflammatory Infiltrate	Congestion	Desquamation	Necrosis	Edema	Inflammatory Infiltrate
	(%)					(%)
Without AGPs	1.25 ^a^	2.63 ^a^	2.38 ^a^	1.25 ^a^	0.50 ^a^	1.13 ^a^
With AGP	0.13 ^c^	1.63 ^bc^	0.38 ^c^	0.00 ^b^	0.00 ^b^	0.13 ^b^
AGP + prebiotics 1 and 2	1.00 ^ab^	1.13 ^c^	1.25 ^bc^	0.00 ^b^	0.00 ^b^	0.63 ^ab^
AGP + prebiotic 1	1.00 ^ab^	1.63 ^bc^	1.25 ^bc^	0.00 ^b^	0.00 ^b^	0.25 ^b^
AGP + probiotic	0.75 ^ab^	2.00 ^ab^	1.75 ^ab^	0.00 ^b^	0.00 ^b^	0.00 ^b^
AGP + essential oil	0.13 ^c^	1.88 ^ab^	1.13 ^bc^	0.13 ^b^	0.38 ^a^	0.00 ^b^
AGP + organic acid	0.63 ^bc^	2.38 ^ab^	2.50 ^a^	0.50 ^b^	0.38 ^a^	0.00 ^b^
Probability	0.0001	0.0001	0.0001	0.0001	0.0058	0.0003

AGP: Antibiotic growth promoters. a-b-c: Means followed by different letters in the columns are distinguished by the Kruskal–Wallis test at 5% probability level. Prebiotic 1: prebiotics with a yeast cell wall, yeast extract, fructooligosaccharides, and galactooligosaccharides. Prebiotic 2: prebiotic composed of enzyme-hydrolyzed *Saccharomyces cerevisiae*, yeast wall and extract, 1.3 and 1.6 beta glucans, and mannan oligosaccharides.

**Table 13:** Indexes are shown inside the ileum of broilers according to the treatments at 6 and 21 days of age.

Treatments	6-Day-Old					21-Day-Old				
	Inflammatory Infiltrate	Congestion	Desquamation	Necrosis	Edema	Inflammatory Infiltrate	Congestion	Desquamation	Necrosis	Edema
	(%)					(%)				
Without AGPs	1.50 ^a^	2.50 ^a^	3.00 ^a^	1.25 ^a^	0.25 ^ab^	0.88 ^a^	1.38 ^ab^	1.50 ^ab^	0.50 ^a^	0.38 ^b^
With AGP	0.00 ^c^	1.25 ^c^	0.63 ^c^	0.00 ^b^	0.38 ^ab^	0.00 ^b^	1.00 ^b^	0.50 ^b^	0.00 ^b^	0.00 ^b^
AGP + prebiotics 1 and 2	1.00 ^ab^	1.50 ^bc^	0.63 ^c^	0.00 ^b^	0.50 ^ab^	0.75 ^ab^	1.63 ^ab^	1.13 ^ab^	0.13 ^b^	0.25 ^b^
AGP + prebiotic 1	1.25 ^ab^	1.75 ^bc^	1.13 ^bc^	0.00 ^b^	0.00 ^b^	0.25 ^ab^	2.00 ^a^	1.25 ^ab^	0.00 ^b^	0.25 ^b^
AGP + probiotic	0.88 ^b^	2.00 ^ab^	2.38 ^a^	0.00 ^b^	0.13 ^ab^	0.13 ^b^	1.50 ^ab^	0.75 ^b^	0.00 ^b^	0.00 ^b^
AGP + essential oil	0.63 ^b^	2.00 ^ab^	1.38 ^bc^	0.00 ^b^	0.63 ^ab^	0.00 ^b^	1.50 ^ab^	1.13 ^ab^	0.00 ^b^	0.88 ^a^
AGP + organic acid	0.63 ^b^	2.13 ^ab^	1.63 ^b^	0.25 ^b^	0.75 ^a^	0.00 ^b^	2.00 ^a^	2.00 ^a^	0.00 ^b^	0.57 ^ab^
Probability	0.0001	0.0001	0.0001	0.0001	0.0149	0.0020	0.0028	0.0274	0.0226	0.0005

AGP: Antibiotic growth promoters. a-b-c: means followed by different letters in the columns are distinguished by the Kruskal–Wallis test at 5% probability level. Prebiotic 1: prebiotics with a yeast cell wall, yeast extract, fructooligosaccharides, and galactooligosaccharides. Prebiotic 2: prebiotic composed of *Saccharomyces cerevisiae* hydrolyzed by enzymes, yeast wall and extract, 1.3 and 1.6 beta glucans, and mannan oligosaccharides.

## Data Availability

The data supporting the reported results of this study will be made available upon request to the corresponding author.
